# Comparison between two solute equations and bioimpedance for estimation of body fluid volumes

**DOI:** 10.1186/s40635-022-00436-9

**Published:** 2022-03-07

**Authors:** Robert G. Hahn, Marc Giménez-Milà

**Affiliations:** 1 Research Unit, Department of Anesthesia & Intensive Care, Södertälje Hospital, 152 86 Södertälje, Sweden; 2 Karolinska Institutet at Danderyds Hospital (KIDS), Stockholm, Sweden; 3 Department of Anaesthesiology, “CLINIC de Barcelona” Hospital, University of Barcelona (UB), Carrer Villaroel 170, 08036 Barcelona, Spain; 4 Systems Pharmacology Effect Control and Modeling (SPEC-M) Research Group, “CLINIC de Barcelona” Hospital, Barcelona, Spain; 5 “Institut d’Investigacions Biomèdiques August Pi i Sunyer (IDIBAPS)”, Barcelona, Spain

**Keywords:** Fluid exchange, Bioimpedance analysis, Crystalloids, Mass balance equations

## Abstract

**Background:**

The extracellular volume (ECV) and intracellular volume (ICV) estimated by bioimpedance analysis (BIA) deviates markedly from the textbook volumes of 20% and 40% of the body weight (BW). We estimated the transcellular exchange of water by calculating solute equilibriums after fluid challenges to examine whether the BIA or the textbook volumes are likely to be most correct.

**Methods:**

Data was retrieved from 8 healthy male volunteers who received 25 mL/kg of Ringer’s solution or 3–5 mL/kg of hypertonic (7.5%) saline over 30 min after the ECV and ICV had been estimated by BIA. The exchange of water between the ECV and the ICV was calculated according to a sodium equation and an osmolality equation. Simulations were performed, where deviating body fluid volumes were applied.

**Results:**

The mean ECV measured with BIA was 24.9% of BW (*p* < 0.05 versus the “textbook” volume). Mean ICV measured with BIA was 22.3% of BW (*p* < 0.05). The sodium and osmolality equations correlated closely with respect to the translocation of water across the cell membrane (*r*^2^ = 0.86). By applying the “textbook” ECV, the sodium equation indicated that Ringer’s solution exchanged negligible amounts of water, while hypertonic saline withdrew 1.4 L from the ICV to the ECV. By contrast, applying the BIA-derived ECV to the sodium equation implied that 3 L of water would be translocated from the ECV to the ICV once hypertonic saline was administered.

**Conclusion:**

The “textbook” ECV and ICV volumes but not the BIA-derived volumes were consistent with the fluid shifts obtained by two solute equations.

## Introduction

The body fluids are traditionally divided into an extracellular volume (ECV) and an intracellular volume (ICV). Medical textbooks teach that the ECV represents 20% of the body weight (BW), while ICV corresponds to 40% of the BW based on indicator–dilution techniques [1–3].

These fractions have been challenged by studies, where electric bioimpedance analysis (BIA) has been applied. The software used with the BIA is calibrated using isotope methods [4–6] but the obtained values differ from the “textbook volumes” [7]. The typical finding with BIA is that the ECV is larger than 20% and that the ICV is smaller than expected, making the ECV:ICV ratio to be 1:1.5 instead of 1:2 [8–12]. It is unclear whether the textbook volumes or the BIA-derived volumes are correct.

In the present work, we study the usefulness of mass balance equations to examine whether a reported body fluid size is reasonable or more likely to be erroneous. For this purpose, we applied a sodium and an osmolality equation that are dependent on correct sizes of the ECV and ICV to estimate the transcellular shift of water after a fluid challenge [13, 14]. The composition and amount of fluid administered determine direction and volume of fluid exchange, which are fairly well known for the literature. However, the relevance of using fluid shift calculations as a quality control of reported body volumes has never been studied.

A comparison between the “textbook volumes” and those reported by BIA served as an example. We used data obtained during experiments in volunteers, where BIA had been measured and isotonic and hypertonic electrolyte fluids were infused in volunteers [14, 15].

## Methods

The report is a retrospective analysis of two prospective studies of hemodilution during fluid therapy in volunteers. The protocols had been approved by the Ethics Committee of Huddinge University Hospital (Dnr 228/98 and 54/95, Chairmen Ola Eiken and Lennart Kaijser) [14, 15]. Informed consent was obtained from all participants. Data was treated according to confidentiality guidance. The studies originally comprised 80 infusions of both isotonic and hypertonic fluids, but we only used the subset of 15 experiments collected when the 8 volunteers were euvolemic and all data necessary for the evaluation was present.

### Procedure and measurements

The volunteers had a light breakfast consisting of one glass of water or milk and one sandwich at least 2 h before the infusion. They voided and were weighed just before the infusion started. A recumbent equilibration period of 30 min was allowed before the experiments were initiated.

The infusions were 20–25 mL/kg of nearly isotonic crystalloid fluid either Ringer’s lactate or acetate (*Ringer group*) or 5 mL/kg of 7.5% (hypertonic) saline or 3 mL/kg of 7.5% saline in 6% dextran (*Hypertonic group*). All infusions were administered at a constant rate over 30 min using infusion pump. Volunteers received both types of infusion. The washout period between the experiments was at least 1 week.

Blood samples were drawn just before the infusions started, at the end of the infusions (at 30 min), and at 240 min. They were analyzed for the serum sodium and the serum osmolality at the hospital’s certified clinical chemistry laboratory. The urinary excretion was measured at 240 min and taken into consideration for sodium and osmolality equation calculation at that point in time. Hence, any adjustment of the fluid shifts indicated by the osmolality equation at 30 min and 240 min was solely due to urinary losses of water and osmolality. By contrast, the sodium equation considered measurements of serum sodium both at 30 min and 240 min in addition to urinary losses at 240 min.

Sodium was analyzed by an ion-selective, direct potentiometry technique using an IL BGE analyzer (Instrumentation Laboratory, Milan, Italy) with a coefficient of variation of between 1 and 2%. Osmolality was measured by an Osmometer 3C2 (Advanced Instruments Inc., Norwood, MA) which is based on freezing-point depression. The CV was approximately 1%.

The size of the ECV and the ICV were estimated by multi-frequency bioelectrical impedance (Xitron 4000B Bioimpedance Spectrum Analyzer, Xitron Technologies Inc., San Diego, CA) just before each infusion was initiated. The software calculates these volumes based on a series of 50 currents of different frequencies between electrodes affixed to the dorsum of one hand and one foot. The mean of three consecutive measurements was used.

### Calculations

The diffusion of fluid into and out of the cells (fluid shift) was calculated by both a sodium equation and an osmolality equation.

The sodium equation is based on a mass balance concept implying that sodium ions (Na) and water in the ECV remain constant over time except for additions and losses. All these additions and losses are known except for fluid exchange between the ECV and ICV spaces. Since Na is distributed throughout the ECV space, the serum Na concentration at any time (*T*) during or after an intravenous infusion of fluid (Na_*T*_) equals the amount of Na in the ECV volume divided by the current ECF volume.

The serum concentration before the intervention, Na_o_, and at any time *t* after it has occurred, Na_*T*_ are then connected in the following equation [13]:

$${\text{Fluid exchange}} = {\text{ECV}}_{{\text{o}}} + {\text{infused volume}}{-}\left[ { \, {{\left( {{\text{Na}}_{{\text{o}}} {\text{ECV}}_{{\text{o}}} + {\text{infused Na}}} \right)} \mathord{\left/ {\vphantom {{\left( {{\text{Na}}_{{\text{o}}} {\text{ECV}}_{{\text{o}}} + {\text{infused Na}}} \right)} {{\text{Na}}_{T} }}} \right. \kern-\nulldelimiterspace} {{\text{Na}}_{T} }}} \right]$$

If urinary excretion has occurred, the voided volume should be subtracted from the infused fluid volume. Similarly, the sodium ions excreted in the urine should be subtracted from the infused amount of sodium. The positive and negative signs in the sodium equation were inverted to enable comparison between equations.

The osmolality equation is built on the fact that the osmolality is always the same in the ECV and ICV. Hence, we can derive [14]:

$$\frac{{{\text{ECF}} \cdot {\text{S-osmolality}}\;{\text{start}} + {\text{infused}}\;{\text{osmoles}}}}{{{\text{ECF}} + {\text{fluid}}\;{\text{exchnage}} + {\text{infused}}\;{\text{volume}}}} = \frac{{{\text{ICF}} \cdot {\text{S-osmolality}}\;{\text{start}}}}{{{\text{ICF}} - {\text{fluid}}\;{\text{exchange}}}}$$

This mass balance equation implies that the amount of solutes divided by the fluid volume must remain the same after manipulation of any of involved factors. The only unknown factor in this equation is “fluid exchange” if we assume that the ECV amounts to 20% of the BW and ICV to 40% of the BW. Note that no second blood sample is needed. A positive sign in fluid shift of both equations indicates transfer from ICV to ECV.

### Statistics

Data is presented as the mean and standard deviation (SD). The relationship between fluid shift according to different equations was evaluated by simple linear regression analysis, where *r*^2^ = coefficient of determination. The Student’s *t* test was used to compare the “text-book” body fluid volumes with those obtained by BIA. Data analysis was carried out using the R statistical programming environment (version 3.5 for windows, R Core Team, 2015). *p* < 0.05 was considered statistically significant.

## Results

### Demographic and body fluid data

A complete data set was obtained from 15 experiments that were performed in eight male volunteers with a mean age (SD) of 32 (8) years and with a BW of 82 (9) kg. Three volunteers received Ringer’s lactate and three Ringer’s acetate (*Ringer group*), while 7.5% saline was given to two individuals and 7.5% saline in 6% dextran 70 dextran to seven volunteers (*Hypertonic group*).

The ECV measured with BIA was 20.4 L (24.9% of BW), which is 4 L more than given by the widely assumed fraction of 20% of the BW (*p* < 0.05). Mean ICV measured with BIA was 18.5 L (22.3% of BW), which is significantly smaller than the assumed 32.5 L based on 40% of BW (*p* < 0.05).

Table 1 depicts the measured and theoretically assumed ECV and ICV for each experiment and fluid group.

**Table 1:** Body weight, BIA, and theoretically assumed ECV and ICV for each experiment and fluid group

	Body weight (kg)	BIA ECV (L)	BIA ICV (L)	Assumed ECV (L)	Assumed ICV (L)
*Hypertonic group*					
1	78	23.5	20.3	15.6	31.2
2	70	18.1	17.2	14	28
3	95	23.3	21.5	19	38
4	72	18.1	16.6	14.4	28.8
5	90	18.8	15.3	18	36
6	88	21	16	17.6	35.1
7	76	19.4	16.1	15.2	30.4
8	72	17.6	17.5	14.4	28.8
9	79	19.8	19.6	15.8	31.6
Mean (SD)	81.9 (10.1)	19.9 (2.2)	17.7 (2.1)	16 (1.7)	31.9 (3.5)
*Ringer group*					
10	93	23	20.38	18.6	37.2
11	93	22.6	20.25	18.6	37.2
12	80	21.9	20.58	16	32
13	80	21.3	21.02	16	32
14	72	17.6	17.8	14.4	28.8
15	79	20.6	18.04	15.8	31.6
Mean (SD)	82.8 (8.0)	21.2 (1.9)	19.6 (1.3)	16.5 (1.6)	33.1 (3.3)
Mean of both groups (SD)	82.1 (8.8)	20.4 (2.2)	18.5 (2.0)	16.2 (1.7)	32.4 (3.4)

Table 2 shows the infused and excreted amounts of fluid and electrolytes for each experiment and fluid group. The mean (SD) initial serum osmolality was 293.0 (2.2) mOsm/kg and serum sodium 140.8 (1.7) mmol/L. The mean volume (SD) infused in the Ringer group was 1.95 (0.08) L and in the hypertonic group 0.28 (0.08) L. The hypertonic infusions administered 37% more sodium than the Ringer group, but the excretion differed by 110% (*p* = 0.002). Similarly, 87% more osmoles were excreted compared to the Ringer group (*p* < 0.01), while the excreted fluid volumes did not differ (*p* = 0.39).

**Table 2:** Infused and excreted amounts of fluid and electrolytes for each experiment and fluid group

	Infused volume (mL)	Infused osmoles (mOsm)	Infused sodium (mmol)	Excreted volume (mL)	Excreted osmoles (mOsm)	Excreted sodium (mmol)
*Hypertonic group*						
1	234	600	296	750	595	213
2	210	539	266	700	606	210
3	285	731	361	800	586	183
4	216	554	274	240	188	63
5	270	693	342	850	501	190
6	228	585	289	950	536	181
7	440	1128	556	900	656	190
8	360	924	456	400	374	118
9	237	608	300	975	712	205
Mean (SD)	275 (77)	707 (198)	348 (98)	726 (269)	528 (160)	173 (50)
*Ringer group*						
10	1975	543	257	1100	306	87
11	1975	543	257	300	133	38
12	2000	550	260	1100	329	78
13	2000	550	260	600	123	53
14	1800	495	234	900	455	156
15	1975	543	257	1200	347	79
Mean (SD)	1950 (716)	537 (21)	254 (10)	866 (350)	282 (130)	82 (41)

### Fluid transfer across the cell membrane

The osmolality equation indicated that the transfer of water across the cell membrane in the Ringer group amounted to − 72 (10) mL at 30 min and to − 183 (174) mL at 240 min. The corresponding values according to the sodium equation were − 194 (9) mL and + 198 (104) mL, respectively.

In the Hypertonic group fluid recruitment derived by the osmolality equation was + 1355 (595) mL at 30 min and + 694 (404) mL at 240 min. When based on the sodium equation the fluid transfer amounted to + 1212 (385) mL and + 1178 (466) mL.

The urinary losses of solutes and water were considered in the calculation at 240 min but these data were not available at 30 min.

**Fig. 1:**
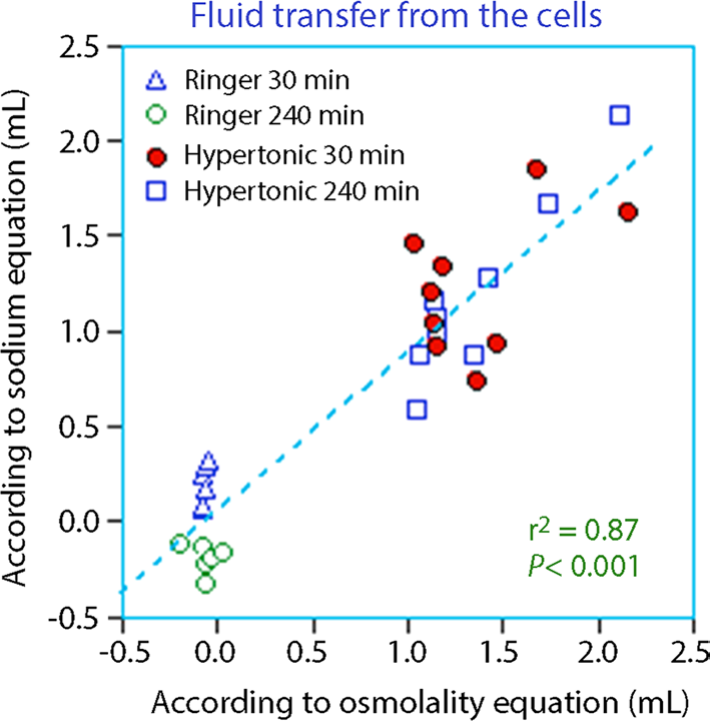
Calculation of the recruitment of fluid from the ICV to the ECV at 30 min and 240 min after infusion of Ringer’s solution and hypertonic saline in volunteers. Each point is one measurement (two in each volunteer). No correction for urinary excretion was performed

Figure 1 illustrates how the estimated fluid transfer compares between the two equations when based on the assumed ECV and ICV of 20% and 40% of BW, respectively.

**Fig. 2:**
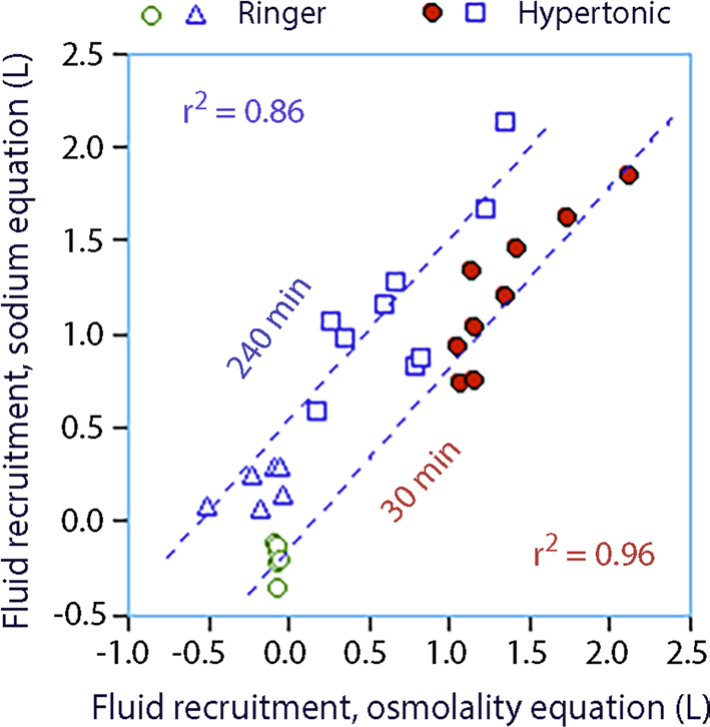
Same plot as Fig. 1 but with correction of excreted fluid, sodium and osmolality at 240 min. Such data were not available at 30 min. The left displacement of the curve at 240 min as compared to Fig. 1 shows the influence of urinary excretion on the results

Figure 2 illustrates the displacement of the data that occurs when the excreted fluid, sodium, and osmolality has been incorporated into the equations at 240 min (no measurement of losses are available at 30 min).

The mean difference between the output of the osmolality and the sodium equation was − 153 mL (SD 350 mL).

### Simulation of fluid exchange with altered body fluid volumes

The following simulations aimed to examine whether the solute data best agreed with ECV and ICV volumes as measured by the BIA or by the commonly reported “textbook” fractions of 20% and 40% of the BW.

**Fig. 3:**
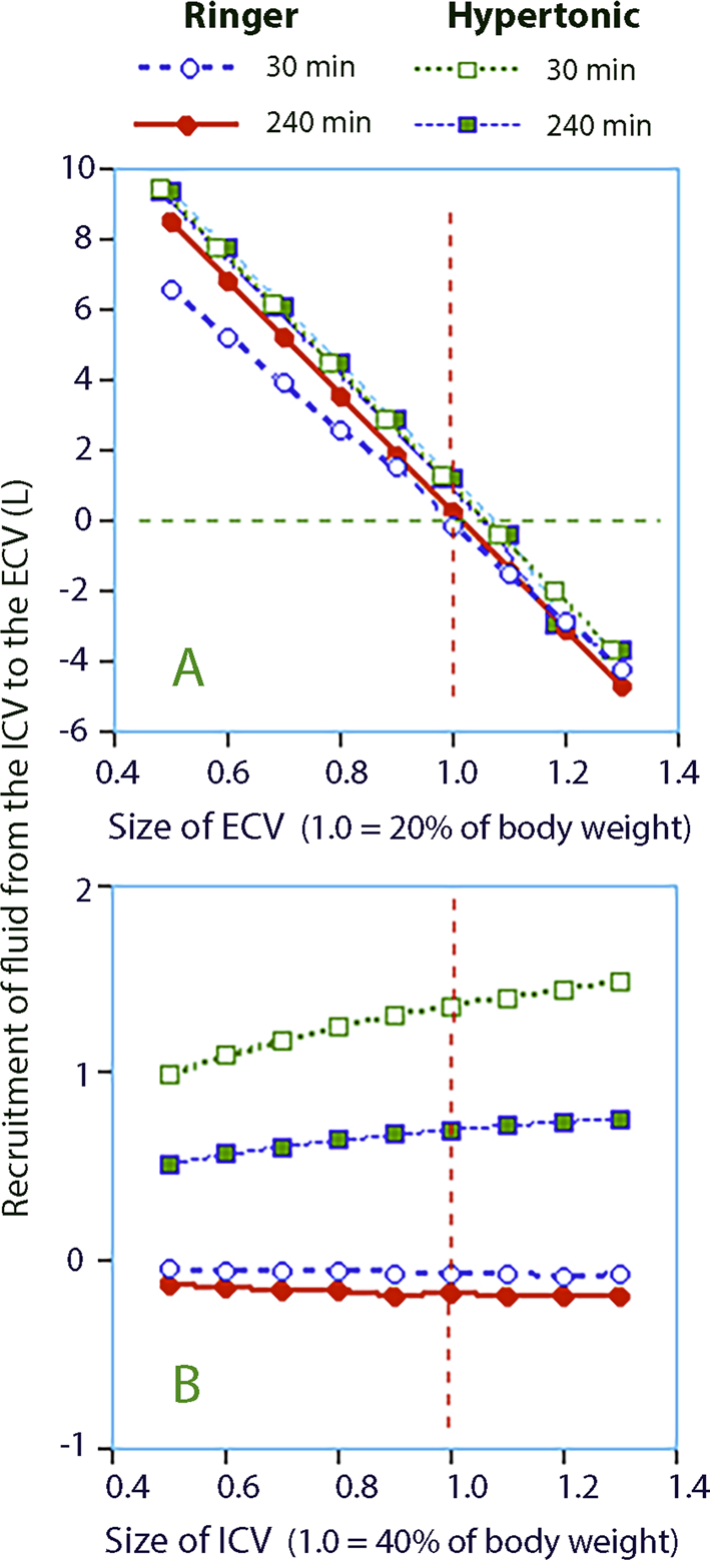
Transfer of fluid from the ICV to the ECV as estimated by the **A** Sodium equation and **B** Osmolality equation. The data points were obtained by setting ECV and ICV to arbitrary volumes that deviate from the “textbook” fractions of 20% and 40% of the body weight with a factor

The sodium and osmolality equations were then used to simulate the transcellular fluid shift based on changes of assumed ECV and ICV volumes in steps of 10% between 50 and 130% of the “textbook” fractions. These simulations showed that the ECV but not the ICV greatly affected the calculated fluid shift (Fig. 3A, B).

As reported above, the osmolality equation indicated that 694 mL of intracellular fluid had been recruited at 240 min in hypertonic group when ICV was set to 40% of the BW. The same fluid shift would be obtained by the sodium equation if the ECV had been 20.6% of the BW. With that ECV size, the fluid recruitment of hypertonic fluid became virtually identical at 688 mL.

On setting the ECV to 1.2 (25% of BW) in the sodium equation (Fig. 3A) the fluid exchange in Ringer and hypertonic groups indicate that approximately 3 L of fluid would be transferred *from* the ECV *to* the ICV, i.e., in the direction being contrary to the expected one. Therefore, according to our simulation, the BIA did not provide a truthful estimate of the ECV.

**Fig. 4:**
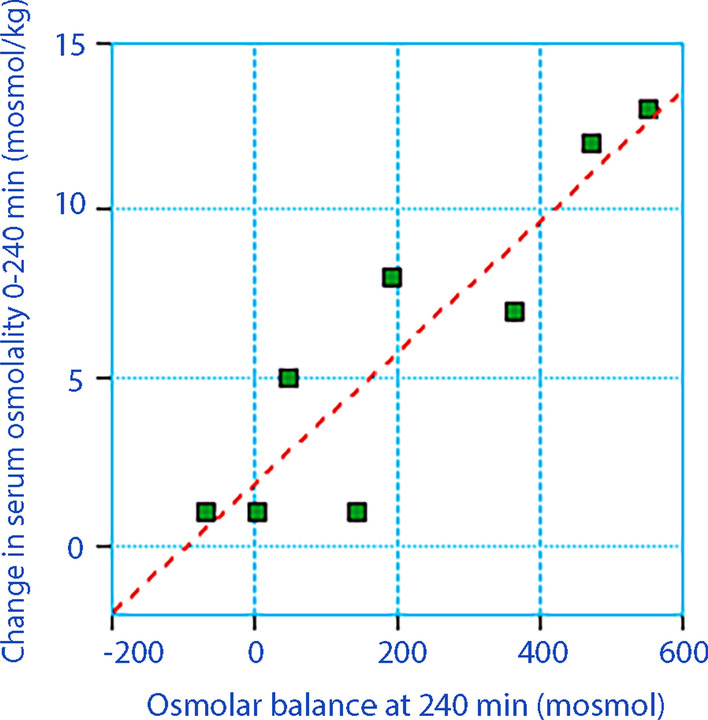
Osmolar balance (= difference between the infused and excreted osmolality) versus the increase in serum osmolality in the hypertonic group. One extreme outlier is not shown. The Total Body Water (= sum of ECV and ICV) is calculated as *x*/*y*

Deliberate changes of the ICV in the osmolality equation resulted in much smaller variations of the fluid translocation (during this simulation, the ECV was maintained at 20% of the BW, Fig. 3B). The slope obtained by assuming different values of ICV was not steep enough for reaching a reasonably firm conclusion about the size of the ICV. Instead, we plotted the osmolar balance at 240 min versus the change in serum osmolality for the hypertonic infusions to obtain a figure of the size of the ICV. This regression showed that adding 600 mosmol to the body corresponded to a rise in serum osmolality of 13.8 mosmol/kg (Fig. 4). The volume of distribution of the administered osmolality was then 600/13.8 = 43.5 L. The body weight of these volunteers averaged 82 kg, which yields that the total body water occupied 53% of the BW. With an ECV volume being 20% of the body weight the size of the ICV was 33% of the BW, or 27 L.

## Discussion

### Key result

Our results illustrate that solute equations can be applied after an infusion of isotonic or hypertonic fluid to ascertain whether assumed or measured ECV is reasonable from a physiological point of view. The osmolality equation tells us that Ringer’s should translocate only very minor amounts of water across the cell membrane. However, using the solute equations with an ECV that differs that from the “textbook” value it would imply that a large transfer of water in and out of the ECV occurs, which we know is not the case (Fig. 3A). The law of osmolality also tells us that hypertonic saline translocates water from the ICV to the ECV assuming textbook fluid compartments. Nevertheless when the solute equations are used they indicate a flow from ECV to ICV if we apply the BIA-derived ECV, which is an aberrant fluid dynamic.

The precise volumes may be modified by urinary excretion and solutes and fluid, but our calculations show that dramatically different flows are predicted if the ECV is changed. By contrast, the solute equations proved less useful to ascertain whether a reported ICV is reasonable.

### Transcellular recruitment of water

The transcellular transport of fluid as indicated by of one sodium and one osmolality equation was calculated in a young volunteer population given an infusion of hypertonic or isotonic crystalloid infusion. Both equations yielded similar mean values for transcellular transport of water when the ECV and ICV were set to the “textbook” volumes. The Ringer infusions caused only minor changes of intracellular water, while hypertonic fluid withdrew more than 1 L from the ICV, which represents approximately 4.5 times the infused volume of 7.5% saline. One could perhaps expect 7.5 times the infused volume would be withdrawn, but more pronounced transcellular allocation of water is counteracted by concentration of the intracellular electrolytes.

No data on the urinary excretion was obtained at 30 min, but losses of electrolytes and water were probably quite small at that time. Complete data was only available at 240 min. Then calculations then disclosed a left-sided displacement of the fluid recruitment curve as evidence that some fluid had already returned to the ICV (cf. Figs. 1 and 2).

### The ECV volume

Our results suggest that calculations based on solute changes can be used to examine how realistic a reported ECV volume is. Logical transcellular fluid shifts were obtained when the ECV was set to the “textbook volumes” being 20% of BW for the ECV and the 40% of the BW for the ICV. However, the simulation using the BIA-derived size the ECV is highly unlikely to be correct. Being 25% of the BW would mean that several liters of fluid enters the ICV when hypertonic saline is infused (Fig. 3A). The law of osmolality rather tells us that hypertonic saline recruits fluid from the ICV, and not the opposite.

We have previously pointed out that the ECV and ICV measured with BIA disagrees with these commonly accepted “textbook” volumes, although it has been unclear which one is most correct [7]. The BIA volumes we reported here, suggesting that the ECV accounted for 25% of the BW and that the ICV accounted for 22% of the body weight, are not unique but reasonably in line with previously published data [4–6, 8–11, 14]. However, our present data support that the “textbook” ECV is the more likely to be correct than the BIA values.

### The ICV volume

Our results further show that calculations based on solute changes cannot be used to examine how realistic a reported ICV volume is using the same reasoning as for the ECV volume. The size of the ICV could not be adequately assessed by the osmolality equation. However, a regression plot comparing the osmolar balance and the change in serum osmolality yielded more clear information. This plot indicated that the ICV amounted to 33% of the BW (Fig. 4). Hence, the osmolality still obtained an ICV that was smaller than the conventionally assumed 40% of the BW, although not as small as the volume obtained with the BIA method.

### Why do the volumes differ?

There may be several explanations to the difference in body fluid volumes between the textbooks and the more recently obtained BIA values. One is that the ICV, and to some degree the ECV, depends on the daily water intake as indicated by urinary biomarkers; a low intake is associated with a larger ICV and a high daily intake, resulting in more diluted urine, with a smaller ICV [12]. The large “textbook” size of the ICV might then have been derived from a cohort with a low daily intake of water. However, our present study does not support that view as measurements from the same individuals were compared. The electrolyte data suggest that that the size of ECV is likely to have been approximately 20% rather than 25%, while the ICV is probably somewhat lower than 40%, even in young men.

Alternatively, the radioisotope measurements used to create the computer program that converts the BIA spectral signals to ECV and ICV for the widely used Xitron spectroscope may have been interpreted erroneously. The original study used for this purpose measured the ECV with bromide and arrive at 24.5% of the BW [5], which is 5% higher than our results with the same method [16]. The authors deliberately allowed BIA to underestimate the ICV as indicated by deuterium by as much as 7 L, because they relied more on measuring ICV by radioactive potassium [5]. In the present study, we found the difference between BIA and the ICV indicated by our electrolyte calculations to be the same at 7.4 L. This suggests that the deuterium volume instead of the radioactive potassium volume might have been more appropriate to used for transforming the BIA signals to volumes.

### Literature

The BIA literature has caused uncertainty about the sizes of the body fluid compartments. Several previously published studies using BIA report fractions higher than the commonly accepted 20% of the BW for ECV and markedly lower fractions than 40% for the ICV. These results seem to be in line with findings in this study with ECV accounting for 25% of BW and ICV for 22% of BW of BW (Table 1).

When applying BIA in males, Ohashi et al. found the ECV to be 22.4% of the BW [11], while in women showed to be approximately 22% [12]. Other studies have also reported ECV volumes several percentages higher than 20% [8, 10, 17] and the ICV to be around 20% of the BW [7, 8, 18].

Old studies using dilution–indicators techniques report body fluid volumes that agree better with the “textbook volumes” than the more recent BIA literature [2, 3, 19]. One study in volunteers from 2005 yielded 18.3% of the BW when estimating the ECV by sodium dilution, 19.6% by iohexol dilution, and 20.5% by bromide [16].

The presence of fractions of structured water may possibly explain differences on measuring body compartments with different methods [20, 21]. Norberg et al. used ethanol and deuterium oxide to calculate TBW and found a bias of − 12.6% which the authors explained as being due to measurement of different water spaces [19].

Sodium might the stored in non-osmotic form when given in a large surplus. Engberink et al. showed that only 47% of the sodium and 55% of the potassium could be recovered in the urine 4 h after infusion of hypertonic saline in 12 healthy volunteers [22]. Possible structures for accumulation of the non-osmotic sodium might be the glycosaminoglycans present in skin, bones and cartilages. However, the time frame for the conversion of ionic to non-ionic sodium is unclear. Our data confirms that 50% of the infused sodium is recovered in the urine after 4 h but interpreted the remaining sodium as an osmotic force that recruited fluid from the ICV, which by itself diluted the plasma sodium level.

## Limitations

The sample size was relatively small and did not include females, who are known to have a different water composition than men. A surgical population of elderly patients with comorbidities could also have had different body fluid composition and fluids [23, 24].

Some of the hypertonic infusions contained dextran, which redistributes fluid from the interstitial fluid space to the plasma. However, the osmolality due to the colloid component is negligible, leaving almost no room for additional redistribution of fluid across the cell membrane.

## Conclusions

Mass balance calculations based on whole-body equilibriums of solutes after intravenous infusion of isotonic and hypertonic fluid show that the estimated water exchange across the cell membrane is incompatible with the size of the ECV obtained by Xitron BIA. By contrast, the same calculations agree well with the ECV volume amounting to 20% of the body weight which is commonly reported in medical textbooks.

## Data Availability

Data can be available on request.

## Abbreviations

BIA: Bioimpedance analysis
BW: Body weight
ECV: Extracellular volume
ICV: Intracellular volume
